# Detection of Sleep-Disordered Breathing in Patients with Spinal Cord Injury Using a Smartphone

**DOI:** 10.3390/s21217182

**Published:** 2021-10-29

**Authors:** Yolanda Castillo-Escario, Hatice Kumru, Ignasi Ferrer-Lluis, Joan Vidal, Raimon Jané

**Affiliations:** 1 Institute for Bioengineering of Catalonia (IBEC), Barcelona Institute of Science and Technology (BIST), 08028 Barcelona, Spain; iferrer@ibecbarcelona.eu (I.F.-L.); rjane@ibecbarcelona.eu (R.J.); 2 Department of Automatic Control (ESAII), Universitat Politècnica de Catalunya-Barcelona Tech (UPC), 08028 Barcelona, Spain; 3 Centro de Investigación Biomédica en Red de Bioingeniería, Biomateriales y Nanomedicina (CIBER-BBN), 28029 Madrid, Spain; 4 Fundación Institut Guttmann, Institut Universitari de Neurorehabilitació, 08916 Badalona, Spain; jvidal@guttmann.com; 5 Universitat Autònoma de Barcelona, 08193 Bellaterra, Spain; 6 Fundació Institut d’Investigació en Ciències de la Salut Germans Trias i Pujol, 08916 Badalona, Spain

**Keywords:** spinal cord injury, sleep-disordered breathing, sleep apnea, sleep position, smartphone, biomedical signal processing, mHealth, monitoring

## Abstract

Patients with spinal cord injury (SCI) have an increased risk of sleep-disordered breathing (SDB), which can lead to serious comorbidities and impact patients’ recovery and quality of life. However, sleep tests are rarely performed on SCI patients, given their multiple health needs and the cost and complexity of diagnostic equipment. The objective of this study was to use a novel smartphone system as a simple non-invasive tool to monitor SDB in SCI patients. We recorded pulse oximetry, acoustic, and accelerometer data using a smartphone during overnight tests in 19 SCI patients and 19 able-bodied controls. Then, we analyzed these signals with automatic algorithms to detect desaturation, apnea, and hypopnea events and monitor sleep position. The apnea–hypopnea index (AHI) was significantly higher in SCI patients than controls (25 ± 15 vs. 9 ± 7, *p* < 0.001). We found that 63% of SCI patients had moderate-to-severe SDB (AHI ≥ 15) in contrast to 21% of control subjects. Most SCI patients slept predominantly in supine position, but an increased occurrence of events in supine position was only observed for eight patients. This study highlights the problem of SDB in SCI and provides simple cost-effective sleep monitoring tools to facilitate the detection, understanding, and management of SDB in SCI patients.

## 1. Introduction

Sleeping, like breathing, is an action that we undertake throughout our entire life. We spend approximately 30% of our time sleeping [1], and this is strictly necessary since sleep is the natural state of rest and self-regulation of the organism. However, several diseases can affect sleep quality, producing symptoms of varying severity. These medical conditions, which are called sleep disorders, are highly prevalent in the general population. In recent years, there has been an increasing awareness of the importance of sleep, and sleep epidemiology has become a rapidly growing field [2].

One of the most common sleep disorders is sleep apnea syndrome, also referred to as sleep-disordered breathing (SDB). Sleep apnea is an underdiagnosed medical condition [3,4] that is characterized by repeated episodes of absence (apnea) or reduction (hypopnea) in airflow during sleep, which can be either obstructive or central in origin. These events lead to hypoxia and microarousals, producing sleep fragmentation and leading to symptoms such as excessive fatigue and daytime sleepiness. In addition, evidence suggests that SDB increases the risk of cardiovascular and cerebrovascular diseases [5,6]. The prevalence of moderate-to-severe SDB in the general population is 23% in women and 50% in men, being higher in elderly and obese individuals [7].

Spinal cord injury (SCI) is a condition that causes motor and sensory impairment below the level of the injury but also results in many other health complications. Sleep disturbances are common in patients with SCI and were not usually present before the lesion [8,9,10]. In fact, it has been reported that individuals with SCI have an increased risk of experiencing disrupted sleep [9,11], SDB [11,12,13,14], and daytime symptoms, such as sleepiness and lack of energy [8], even if they can breathe normally when awake [15]. Different predisposing factors contribute to this high incidence of SDB in SCI patients. For instance, SCI can produce significant neuromuscular weakness in the diaphragm, abdominal, and intercostal muscles, thus affecting respiratory function [16]. This is especially critical during sleep, when breathing is completely unconscious, and contributes to the appearance of disturbed sleep patterns. The neuromuscular respiratory weakness seen in SCI has an additional impact on SDB, facilitating the obstruction of the upper airway during sleep and thus hypoventilation [16]. Moreover, the injury can affect both central control of respiration and upper airway collapsibility, thus promoting the appearance of both central and obstructive apneic events [15]. The association of SCI and SDB is complex and may be influenced by multiple factors including the level and completeness of the injury, the time post-injury, and the associated comorbidities [8,15]. For example, it has been reported that patients with tetraplegia are more likely to suffer from SDB than those with paraplegia [17].

Although individuals with SCI are at a higher risk of sleep disorders, sleep quality is rarely evaluated in these patients [15], given the many health complications of SCI and the fact that rehabilitation interventions are mostly targeted at the recovery of motor function. Nonetheless, more efforts should be devoted to the detection and management of sleep disorders in SCI patients, since poor quality of sleep affects the patients’ recovery and well-being and may underlie serious complications. Sleep studies could help to early detect SDB in SCI patients and provide the most appropriate treatment when required. Common screening tools used for sleep apnea detection include self-reported sleepiness [18], questionnaires, such as the Berlin [19] and the STOP-Bang [20] questionnaires, or methods to assess airway dimensions such as the modified Mallampati (MMP) scores [21] or cone-beam computed tomography (CBCT) [22]. However, questionnaires and self-reported symptoms are subjective measures, and the assessment of airway dimensions only provides limited information for sleep apnea detection and can lead to inconclusive results [23]. Currently, the gold-standard technique for sleep evaluation is polysomnography (PSG). However, this technique requires a complex and costly setup, which is very uncomfortable for the patient, who needs to spend the night in the hospital attached to many sensors and wires. Moreover, most clinical sleep laboratories are not prepared to address the needs of patients with SCI such as wheelchair access, special beds, and adequately trained staff [15]. For this reason, simpler cost-effective tools are needed to facilitate the diagnostic procedure and reach more patients. Home respiratory polygraphy may be an alternative to PSG for SDB diagnosis, but stronger evidence is needed and the transport of equipment from the hospital to home and back may create difficulties [24].

In terms of sleep research in SCI individuals, some previous studies have investigated the SDB prevalence in these patients conducting laboratory PSG [11,17], while others relied on portable polygraphy in-hospital [14], home sleep apnea testing [12,25,26,27,28], or pulse oximetry recordings [13,29]. These studies demonstrated significant differences in the number of apneic events between SCI individuals and healthy controls [8], reporting an SDB prevalence in SCI patients that ranged from 15% to 81%. These discrepancies are attributable to different types of patients (e.g., injuries from cervical to lumbar levels), different diagnostic methods, and different criteria for defining SDB [15,16]. More information about these and other studies can be found in some state-of-the-art reviews on sleep research and SDB in SCI [8,15,16,30].

Over the last years, many novel portable systems have been proposed to detect sleep apnea through unobtrusive sensors measuring only a subset of physiological signals such as nasal airflow, thoracic movement, oxygen saturation, or acoustic snoring signals [31]. Recently, our group developed a smartphone-based system for sleep apnea screening, monitoring, and management [32,33,34,35] and compared it with a commercial portable device for sleep apnea diagnosis at home. The proposed system uses the built-in sensors of the smartphone and an external pulse oximeter to acquire acoustic, accelerometer, and pulse oximeter data. These data can be analyzed to detect and quantify apnea and hypopnea events and, thus, stratify patients according to their severity [32,33]. Moreover, smartphone accelerometer data can be used to provide a high-resolution sleep position and investigate its association with apneic events [34,35], since supine position can promote the occurrence of apnea and hypopnea events, a phenomenon known as positional sleep apnea [36,37].

In this work, we aimed to use a smartphone system to perform sleep studies in individuals with SCI, so that we could evaluate SDB and its association with sleep position in these patients using a simple non-invasive mobile health (mHealth) tool. This technical approach has been successfully tested in patients with sleep apnea and healthy subjects in previous studies [32,33,34], but it has never been used in SCI patients. Therefore, the main objectives of this study were to use a novel smartphone system to monitor sleep apnea and sleep position in individuals with SCI and to investigate the characteristics of apnea, hypopnea, and desaturation events in these patients. We hypothesized that SCI patients would have more respiratory problems during sleep than control subjects, which may lead to altered ventilation and oxygenation patterns with an increased number of episodes of flow limitation. The analysis of biomedical signals recorded with a smartphone allowed for us to investigate respiratory events and sleep position in these patients and, thus, to test the potential of this novel mHealth tool to detect respiratory sleep disorders after SCI and its relationship with sleep posture.

## 2. Materials and Methods

### 2.1. Participants

Nineteen individuals with SCI (16 men, 3 women, mean age 43 ± 16 years) were selected to participate in the study (Table 1). The inclusion criteria were cervical or thoracic SCI patients admitted to hospital for rehabilitation, traumatic or non-traumatic in origin, less than 1 year post-injury, and complete or incomplete injuries classified as A–D according to the American Spinal Injury Association Impairment Scale (AIS) [38]. The exclusion criteria were lumbar SCI, no signed consent, previously diagnosed SDB or respiratory disorders, requirement for ventilatory support, pacemaker dependency, arrhythmia and other cardiovascular conditions, any other neurological disorder, and other comorbidities that would contraindicate the test. In addition, nineteen able-bodied subjects (15 men, 4 women) were used as a healthy control group. Control subjects were excluded if they had previously been diagnosed with SDB, other sleep disorders, or any neurological or musculoskeletal disease. The mean age of the control group was 37 ± 14 years with no significant differences between groups (*p* = 0.23). The mean body mass index (BMI) was 23 ± 3 kg/m^2^ in SCI patients and 24 ± 4 kg/m^2^ in controls, without significant differences between groups (*p* = 0.27).

The protocol was approved by the Ethics Committee of the Institut Guttmann (IG code number 2020343) and was conducted in accordance with the Declaration of Helsinki. Written informed consent was obtained from all the participants prior to enrollment.

### 2.2. Data Acquisition System and Experimental Setup

Overnight recordings were obtained from the 19 SCI patients and 19 control subjects using a smartphone system to measure acoustic, accelerometric, and pulse oximetry signals. These data were then analyzed to detect apneic events and monitor sleep position. This allowed for a simplified approach for sleep apnea investigation as described in [32,33,34].

**Figure 1:**
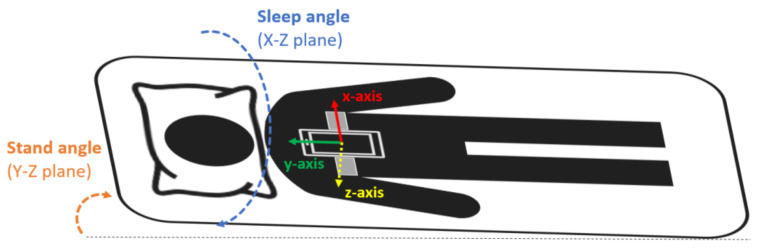
Smartphone placement attached to the subject’s thorax with an elastic band. The orientation of the smartphone accelerometer’s axes and the sleep and stand angles are also indicated.

The smartphone was a Samsung Galaxy S5 SM-G900F with Android 6.0.1. This model was chosen because it is a mid-range phone with a high-quality microphone [39]. Sleep recordings with the smartphone were performed during one full night at the hospital in the SCI patients and at home in the control group. The smartphone was placed and fixed with an elastic band on the subjects’ thorax, over the sternum (Figure 1), in the position suggested by Nakano et al. [40]. In this configuration, the accelerometer’s *x*-axis was in the medial–lateral direction pointing to the left side of the body, the *y*-axis in the inferior–superior direction pointing towards the head of the patient, and the *z*-axis in the anteroposterior direction pointing front to back (Figure 1). During the acquisition, the smartphone was in flight mode with the WiFi and Bluetooth options disabled and the screen switched off.

Control subjects were instructed on how to wear the smartphone system with the elastic band and how to start and stop the acquisition. In the case of SCI patients, due to the fact of their motor disability, the setup was prepared by trained clinical staff. To reduce possible interferences and sound artifacts, subjects slept alone in the bed during the recordings, and they were instructed to try to minimize noise sources such as sounds from machines or electronic devices. Moreover, the smartphone placement ensured that the microphone was close to the nose and mouth. In that position, the signal-to-noise ratio (SNR) of the smartphone recordings was comparable to those of commercial tracheal microphones [39]. Participants were able to choose the sleeping position they liked freely (no specific instructions were given in this regard). Since most of the tetraplegic SCI individuals were unable to turn, their position was changed at least every 3 h during the night by the nursing staff as per clinical protocol.

The proposed mHealth system recorded three signals simultaneously: audio, using the smartphone built-in microphone; tri-axial accelerometry, with the smartphone embedded accelerometer (MPU-6500 sensor); oxygen saturation (SpO_2_), using an EMO-80 wireless fingertip pulse oximeter (EMAY Ltd., Hong Kong, China). The sampling frequency was 48 kHz for audio signals, 200 Hz for accelerometer data, and 1 Hz for SpO_2_. The Android app “Automate” was used to automatically launch the acquisition apps when the phone booted up (Easy Voice Recorder for audio signals and Sensors Logger for accelerometry). Data were automatically stored in the internal memory of the smartphone in .wav and .txt formats, respectively. The pulse oximeter acquired data overnight and was then connected to the smartphone via Bluetooth through the EMAY Pulse Oximeter app to export the SpO_2_ data in .csv files. Total sleeping time had to be at least 4 h, otherwise the examination was repeated.

### 2.3. Signal Processing and Analysis

The analysis of acoustic signals was used to obtain ventilation patterns and detect apneas and hypopneas, while SpO_2_ data allowed for the investigation of oxygenation patterns. Data from the smartphone accelerometer were used to calculate the sleeping position and investigate its relationship with the appearance of apnea and hypopnea events.

Signal processing and analysis was performed offline using custom algorithms developed by our group in MATLAB^®^ r2018a (Mathworks Inc., Natick, MA, USA).

#### 2.3.1. SpO_2_ Analysis

Pulse oximetry recordings allowed us to track the changes in oxygen saturation during the night and, especially, to identify drops in SpO_2_ (i.e., desaturations) caused by apneas and hypopneas.

SpO_2_ values lower than 40% or higher than 100% were considered artifacts and were padded with the previous correct value. The recordings were automatically analyzed to extract a series of features including the awake SpO_2_ (calculated as the median SpO_2_ value in the first 30 s of the recordings), the median and minimum SpO_2_, and the cumulative time spent with SpO_2_ below 90% (CT90) and below 94% (CT94), both expressed as a percentage of the total sleeping time. In addition, the oxygen desaturation index (ODI) was calculated as the number of oxygen desaturations of at least 3% per hour of sleep.

#### 2.3.2. Apnea and Hypopnea Detection

Audio signals were downsampled to 5 kHz, applying a lowpass filter with a cut-off frequency of 2.5 kHz to prevent aliasing. Since there was a lot of wide-band background noise, especially at lower frequencies, spectral subtraction was applied to the signals [41]. An estimated noise model was automatically selected by calculating the root mean squared (RMS) value of each 0.5 s window (99% overlap) in the first 10 min of the recordings, and then joining the 10 windows (non-overlapping with each other) with the lowest RMS to obtain a segment of 5 s to estimate the noise spectrum. After this filtering step, signals were normalized to the maximum absolute value. The first 10 min were discarded for the subsequent analysis. On the other hand, movement artifacts and position changes were detected from accelerometer data [33] and excluded from the analysis, since they also produced sound artifacts.

An entropy-based analysis of acoustic signals was used to detect silence events (SEv) corresponding to apneas and hypopneas as in previous studies [32]. The automatic detection of SEv was based on the calculation of the fixed sample entropy (fSampEn). fSampEn is a measure of time-series complexity, or regularity, that can be used as a robust envelope estimator for noisy physiological signals [42,43]. Being *N* the number of data points in the time series, *m* the embedding dimension, and *r* a tolerance parameter; the fSampEn(*m,r,N*) is defined as the negative natural logarithm of the conditional probability that, in a data set of length N, two sequences that are similar to *m* samples within a tolerance *r* remain similar for *m +* 1 samples [42,43].

The SpO_2_ signal was used to guide the SEv detector, since, to reduce the computational cost and false alarm rate, we only analyzed the audio segments starting 60 s prior to the beginning of each desaturation event and finishing at the end of the desaturation event. Overlapping segments were concatenated up to a maximum length of 10 min. In each of those segments, the envelope of the audio signal was computed by calculating the fSampEn in 0.75 s windows with 50% overlap (*m* = 2, and *r* was equal to the standard deviation of the audio segment). After that, an adaptive threshold was applied; all points below that threshold were found, and regions between 6 and 100 s were selected as SEv [32], corresponding to either apneas or hypopneas.

Once SEv were detected, they were classified into apneas or hypopneas using an algorithm previously published by our group, which showed an accuracy of 82% for apnea/hypopnea classification [32]. The algorithm is based on time–frequency representations of the audio segments to detect low-intensity respiratory sounds and distinguish them from artifacts. If low-intensity respiratory sounds were found, that event was classified as a hypopnea, otherwise it was classified as an apnea. A step-by-step explanation and all the details of the algorithms described in this section for SEv detection and for apnea/hypopnea classification can be found in [32].

The apnea–hypopnea index (AHI) was calculated as the total number of SEv (apneas and hypopneas) per hour of sleep. According to the American Academy of Sleep Medicine (AASM) guidelines [44], subjects can be classified into 4 different categories: normal (AHI < 5), mild sleep apnea (5 ≤ AHI < 15), moderate sleep apnea (15 ≤ AHI < 30), and severe sleep apnea (AHI ≥ 30). After classifying the events, we also calculated the apnea index (AI) and hypopnea index (HI) as the number of apneas or hypopneas per hour of sleep, respectively. Moreover, we calculated the percentage of time spent in apnea and hypopnea events, i.e., the sum of the duration of all the SEv divided by the total time.

#### 2.3.3. Sleep Position Monitoring

From accelerometer data, the sleep and stand angles were derived based on the projection of gravity on the axes of the accelerometer using the algorithms presented in [34,35]. This method was validated in previous studies, showing a 96% agreement with video-validated position from PSG [34].

To remove high-frequency noise, a median filter with a window of 60 s was applied around each accelerometer data sample. Then, the sleep angle was calculated as the angle in the X–Z plane between the accelerometry vector and the (1,0) vector, while the stand angle was calculated as the angle in the Y–Z plane between the accelerometry vector and the (1,0) vector. The sleep angle provides information about the sleep position (lateral rotation) while sleeping. As defined, 0° is a perfect left position, 90° a perfect supine position, ±180° a perfect right position, and −90° a perfect prone position [34,35]. For visualization purposes, the sleep angle was discretized into the 4 classical sleep positions using the thresholds that showed the best agreement with PSG according to previous studies: supine (60–120°), lateral left (−40–60°), lateral right (120–180° and from −180 to −140°), and prone (from −140° to −40°) [34]. The stand angle indicates whether the subject is standing or lying in bed and was used to discard non-lying positions. As defined, ±180° corresponds to a perfect stand position, 0° to a headstand position, and 90° and −90° to a complete lying position.

In addition, we studied and represented the sleep position with angular resolution to investigate its association with the occurrence of apnea and hypopnea events. It is known that some patients with sleep apnea have a higher frequency of events in supine position, a phenomenon that is known as positional sleep apnea. To investigate whether the SCI patients in our sample suffered from positional sleep apnea or, conversely, there was no relationship between the occurrence of events and sleep position, we calculated the percentage of time spent at each sleep angle (i.e., for each sleep angle, θ, in increments of 1°, we computed the percentage of time spent in a 15° window centered at that angle: θ ± 7.5°) and the percentage of events occurring at that angle (i.e., for each sleep angle, θ, we computed the percentage of apneas and hypopneas occurring in sleep angles of θ ± 7.5°) [34]. Then, we compared the percentage of time and the percentage of events occurring at each sleep angle and subtracted the two curves to determine whether more events than expected occurred at each position.

#### 2.3.4. Oral vs. Nasal Breathing

We calculated, for each subject, the percentage of time that was spent breathing through the mouth during the night using an algorithm developed by our group to distinguish between nasal and oral breathing from the spectral characteristics of acoustic breathing signals [32].

Audio signals were segmented into 10 s non-overlapping sliding windows, and each window was classified into nasal or oral breathing. To do so, the fast Fourier transform (FFT) was calculated, and a linear envelope extracted using windows of 15 Hz. While most of the power of nasal breathing is concentrated in the low-frequency band, the spectrum of oral breathing presents a prominent peak between 950 and 2 kHz [32]. Therefore, if the height of the maximum peak in the 950–2000 Hz band was, at least, 60% of the maximum value of the envelope (in the 500–2000 Hz band, to avoid the variable effect of basal noise concentrated at lower frequencies), then the window was labeled as oral breathing and otherwise considered nasal breathing [32]. Once all windows were labeled, we calculated the percentage of windows classified as oral breathing.

### 2.4. Statistical Analysis

The features described in Section 2.3. were extracted for all subjects. Data were also averaged for the SCI and control groups. Means, standard deviations (SDs), and ranges are reported for each group. Mann–Whitney U tests were applied to compare the two groups (SCI vs. control), since normality assumption was not met according to Kolmogorov–Smirnov tests.

In the SCI group, the Spearman correlation coefficient was used to investigate the relationship between the extracted features (especially AHI and SpO_2_ parameters) and age, BMI, injury level, AIS, and time post-injury. An alpha level of 0.05 was used to determine significance for all statistical tests.

## 3. Results

The extracted features are displayed in Table 2 for all SCI patients, while a summary of the values for each group (SCI vs. control) and the *p*-values of the statistical comparisons are presented in Table 3. Below we describe in more detail the results in terms of oxygen saturation, apneas, and hypopneas detected from acoustic signals, sleep position measured from accelerometer data, and prevalence of oral breathing.

### 3.1. SpO_2_ Measures

Table 2 shows the SpO_2_ measures for all SCI individuals, while Table 3 compares the mean values of the SCI and control groups. Mean ODI was 24 ± 14 for the SCI patients (range: 8–60) and 9 ± 7 for control subjects (range: 0.7–27), with significant differences between groups (*p* < 0.001). There were also significant differences in the minimum SpO_2_, CT90, and CT94 (Table 3). However, the awake SpO_2_ and median SpO_2_ did not significantly differ between groups (Table 3). Therefore, although SCI patients had more desaturations of at least 3% than control subjects, the basal SpO_2_ levels before and during sleep were not considerably altered.

### 3.2. Apneas and Hypopneas

**Figure 2:**
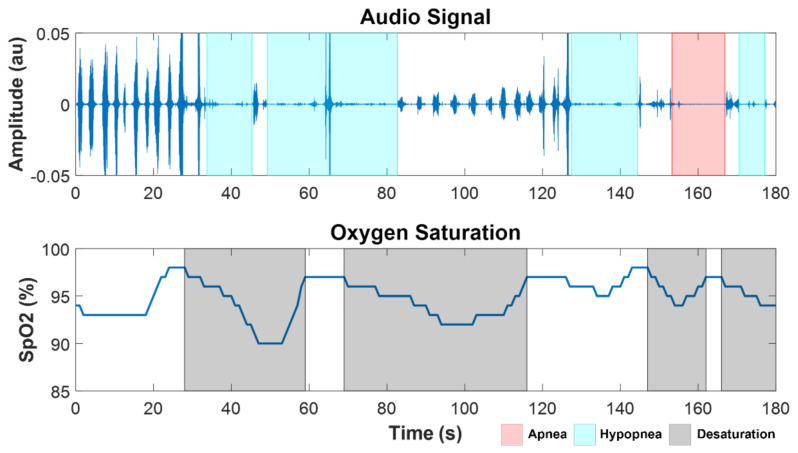
Example of a segment of audio and SpO_2_ signals from a SCI patient with severe OSA (SCI 4, AHI = 60 h^−1^), showing apnea (red), hypopnea (cyan), and desaturation (black) events.

Apnea and hypopnea events were automatically identified from the analysis of acoustic signals and SpO_2_ for all the subjects. An example of a segment of SpO_2_ and audio from the overnight recordings of a SCI patient (SCI 4) with severe SDB (AHI = 60 h^−1^) is shown in Figure 2, with the marks corresponding to automatically detected desaturation, apnea, and hypopnea events.

**Figure 3:**
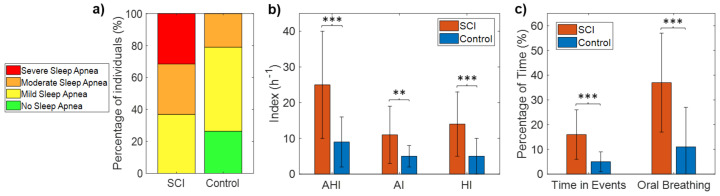
Number of subjects in each category of sleep apnea severity for the SCI and control groups (**a**), mean and SD of AHI, AI, and HI in each group (**b**), and mean and SD of the percentage of time spent during events and the percentage of oral breathing in each group (**c**). Statistically significant differences are indicated with asterisks: ** *p* < 0.01, and *** *p* < 0.001.

Table 2 shows the AHI, HI, AI, and percentage time spent in apnea and hypopnea events for each of the SCI patients. All SCI patients (100%) met the diagnostic criteria for sleep apnea (AHI ≥ 5), with 63% of the patients having moderate-to-severe sleep apnea (AHI ≥ 15). Specifically, none of the 19 patients had AHI < 5, seven patients (37%) had mild SDB (5 ≤ AHI < 15), six patients (31.5%) had moderate SDB (15 ≤ AHI < 30), and six patients (31.5%) had severe SDB (AHI ≥ 30) (Figure 3a, Table 2). In contrast, the incidence of SDB (AHI ≥ 5) in control subjects was 74%, with 21% of the patients having moderate-to-severe SDB (AHI ≥ 15). Specifically, 5 of the 19 controls (26%) had AHI < 5, 10 (53%) had mild SDB (5 ≤ AHI < 15), four (21%) moderate SDB (15 ≤ AHI < 30), and none had severe SDB (AHI > 30) (Figure 3a). Fisher’s exact test confirmed that the occurrence of SDB was significantly higher in the SCI patients than in the control sample (*p* = 0.02).

Mean AHI was 25 ± 15 h^−1^ for the SCI group (range: 8–60) and 9 ± 7 for the control group (range: 0.3–24), with significant differences between groups (*p* < 0.001) (Table 3, Figure 3b). Both AI and HI were significantly higher in the SCI group (Table 3, Figure 3b). The percentage of the total time of the night spent in apnea and hypopnea events was also significantly higher in SCI patients than control subjects (16 ± 10 vs. 5 ± 4, *p* < 0.001) (Table 3, Figure 3c).

### 3.3. Sleep Position

**Figure 4:**
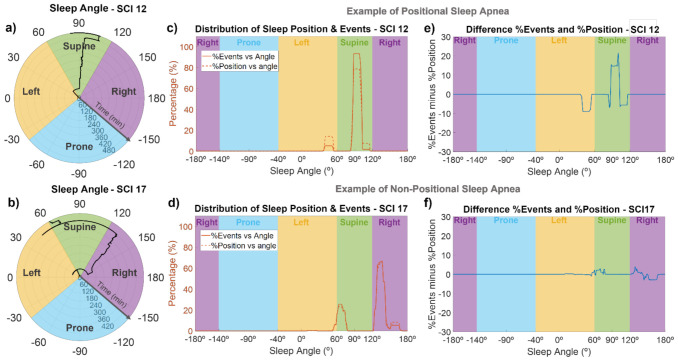
Sleep angle and association between the sleep position and apnea/hypopnea events for two representative SCI patients. The first column (**a**,**b**) are polar plots indicating the sleep angle as a function of time, the middle column (**c**,**d**) shows the percentage of time spent at each sleep angle (dashed line), and the percentage of events occurring during each angle (solid line), and the last column (**e**,**f**) shows the difference between the percentage of time and percentage of events at each sleep angle. SCI 12 (upper panels) is an example of a patient with positional sleep apnea, with an increased frequency of events in supine position, while SCI 17 (lower panels) is an example of a patient with non-positional sleep apnea.

For each subject, the sleep position in angular resolution was computed from accelerometer data. An example of the sleep angle during the night can be seen in the polar plots of Figure 4a,b for SCI 12 and SCI 17. Using these representations, we could monitor the sleep position of each SCI individual throughout the night. For example, as shown in Figure 4a, during the first hour, SCI 12 slept in a left position, close to the border with supine position. After that, his sleep position changed to supine (around 95°), where he spent most of the night, and then, in the 8th hour of sleep, turned slightly to the right up to approximately 105°, to finally reach a sleep angle of around 80° at the very end of the recording. As indicated in Figure 4b, SCI 17 was in a supine position close to the border with lateral left during the first hour, then he changed to a lateral right position for most of the night (with small changes in the sleep angle), and finally moved to a supine position (angles between 60 and 70°), briefly passing through lateral left positions.

The majority of SCI patients slept in supine-like positions. In fact, 10 of the 19 patients slept more than 75% of the time in supine positions, while seven of them alternated between supine and lateral positions, and only one of the patients (SCI13) slept mostly in lateral positions. There was only one patient (SCI 5) who slept in prone position, and it was only for 7% of the time. The sleep positions of the control subjects were more variable, and they slept less in supine position than SCI individuals. Most of the control subjects (10 out of 19) alternated between supine and lateral positions, while six subjects slept predominantly in lateral positions, and only three of them slept predominantly in supine-like positions. Of the total time recorded for the SCI patients, 69% was in supine position, 18% in lateral right, 13% in lateral left, and only 0.4% in prone position. In the case of the control subjects, 40% of the total time was in supine position, 31% in lateral right position, 22% in lateral left, and 7% in prone position.

Figure 4c–f show the association of sleep angle and the occurrence of events for SCI 12 and SCI 17. SCI 12 is an example of a patient with positional sleep apnea (i.e., more events in the supine position), and SCI 17 is an example of a subject without positional sleep apnea. In Figure 4c,d, we can see the percentage of time spent at each sleep angle and the percentage of events occurring at each sleep angle, while Figure 4e,f show the difference between these two curves. SCI 12 is an example of a patient with positional sleep apnea, because almost 95% of events occurred in the supine position (specifically, in sleep angles between 85 and 105°), but less than 80% of the time was spent in this angle range (Figure 4c). In contrast, in left position or sleep angles higher than 105°, the percentage of time spent at those angles was higher (5–10%) than the percentage of events (Figure 4c,e). On the other hand, SCI 17 is an example of non-positional behavior, since the distribution of events and sleep position were very close to each other (Figure 4d), with absolute differences always lower than 4% (Figure 4f), indicating no increased frequency of events in supine position. A positional behavior, similar to that in Figure 4c,e, was observed for 8 of the 19 SCI patients (SCI 1, 3, 4, 8, 10, 12, 16, and 18), without any clear relationship with the injury level or severity. The other patients did not show a clear positional behavior. Regarding control subjects and considering only those with AHI > 5, 9 out of 14 (64%) showed a positional behavior with an increased frequency of apneas and hypopneas in sleep angles corresponding to the supine position.

### 3.4. Oral Breathing

A large amount of mouth breathing while sleeping was observed in SCI patients when compared with control subjects (Table 3, Figure 3c). Oral breathing represented, on average, 37% of the time in SCI patients (range: 9–68%) in contrast to 11% of the time in control subjects (range: 1.3–66%), with significant differences between groups (*p* < 0.001).

### 3.5. Correlation with Age, BMI, and Injury Characteristics

Finally, in the SCI group, we assessed the correlation between the extracted variables and age, BMI, and injury characteristics.

**Figure 5:**
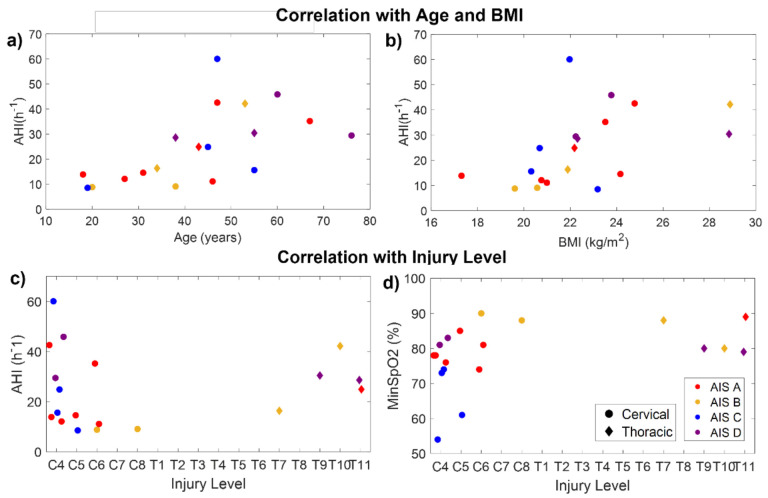
Correlation of AHI with age (**a**), BMI (**b**), and injury level (**c**), and correlation of minimum SpO_2_ with injury level (**d**) for the SCI participants. Colors indicate the injury completeness (AIS level). Circles and diamonds indicate patients with cervical and thoracic injuries, respectively.

Age was significantly correlated with CT94 (r_xy_ = 0.48, *p* = 0.04), CT90 (r_xy_ = 0.66, *p* = 0.002), ODI (r_xy_ = 0.71, *p* < 0.001), AHI (r_xy_ = 0.73, *p* < 0.001), AI (r_xy_ = 0.50, *p* = 0.03), HI (r_xy_ = 0.72, *p* < 0.001), and the percentage time spent in events (r_xy_ = 0.68, *p* = 0.001). Therefore, older patients had more desaturations, apneas, and hypopneas. BMI was significantly correlated with ODI (r_xy_ = 0.71, *p* < 0.001), AHI (r_xy_ = 0.71, *p* < 0.001), HI (r_xy_ = 0.71, *p* < 0.001), and the percentage time spent in events (r_xy_ = 0.61, *p* = 0.005). Figure 5a,b indicate the relationship of AHI with age and BMI in the SCI participants.

The time post-injury was not significantly correlated with any of the outcome measures. No significant correlations were found between AHI and the injury level (Figure 5c). However, considering only the patients with cervical injuries, AHI was significantly correlated with injury level (r_xy_ = 0.58, *p* = 0.03), being higher in patients with higher injuries. Considering the whole sample, the injury level was only found to be significantly correlated with the minimum SpO_2_ (r_xy_ = −0.49, *p* = 0.03), indicating that patients with higher injuries reached lower values of SpO_2_ than those with lower injuries (Figure 5d).

The AHI was not significantly correlated with the injury completeness (AIS level) (*p* = 0.28). However, AIS was correlated with awake SpO_2_ (r_xy_ = −0.70, *p* < 0.001), median SpO_2_ (r_xy_ = −0.66, *p* = 0.002), CT94 (r_xy_ = 0.70, *p* < 0.001), and CT90 (r_xy_ = 0.47, *p* = 0.04). This would indicate that, in our sample, patients with complete injuries had higher SpO_2_ than those with incomplete injuries. Nevertheless, this only happens because age is acting as a confounding factor. As can be seen in Figure 5a, most patients under 40 years of age had complete injuries (AIS A–B), and younger patients tended to have lower AHI. In fact, all SCI patients under 35 years old had mild SDB (AHI ≤ 15), i.e., the lowest severity, even if they had complete high cervical injuries. Something similar occurred with weight: some patients with cervical and complete injuries had milder SDB because they had a very low BMI (Figure 5b).

Finally, the amount of oral breathing was not significantly correlated with age, BMI, injury characteristics, nor any of the other outcome measures (all *p* > 0.27).

## 4. Discussion

In this work, we monitored SDB and sleep position in 19 SCI patients and 19 able-bodied controls from the analysis of acoustic, SpO_2_, and accelerometer signals recorded using a smartphone. We calculated different outcome measures, including SpO_2_ parameters, the apnea/hypopnea index (AHI), or the percentage of oral breathing, which allowed us to study the oxygenation and ventilation patterns during sleep in SCI patients as well as to monitor sleep position and its association with the occurrence of events throughout the night.

The prevalence of SDB in the studied population was very high: all SCI participants (100%) suffered from SDB (AHI ≥ 5). Specifically, according to the AASM guidelines [45], our results indicate that seven SCI patients (37%) had mild SDB, six patients (31.5%) moderate SDB, and six patients (31.5%) severe SDB. This prevalence was much lower in the control sample, despite having a comparable age and BMI: 74% had AHI ≥ 5, a similar SDB prevalence than in the general population [7], with 53% of the subjects having mild SDB, 21% moderate SDB, and none severe SDB.

Our results reflect the alarming incidence of SDB in SCI and are consistent with the previous literature [8,9,15,30]. For instance, using AHI ≥ 15 as the criterion for SDB diagnosis, McEvoy et al. [12] reported a SDB prevalence of 27.5%, while Stockhammer et al. [14] found a much higher value (62%), similar to our result (63%). Both studies used portable monitoring systems to assess SDB in patients with complete and incomplete cervical SCI, more than 6 months post-injury, with a wide range of ages and BMI values. Most of our patients were in the acute state until 6 months after the injury, when the prevalence of SDB seems to be higher than in the chronic state [14]. On the other hand, we found that all SCI participants showed an AHI ≥ 5, which is a higher incidence than in previous works. For example, some authors conducted home sleep apnea tests (polygraphy) in SCI patients, reporting values for the SDB prevalence (AHI ≥ 5) ranging from 40% to 81% [25,27,28], while other studies based on full PSG found that 70–80% of SCI patients had AHI ≥ 5 [17,46]. Discrepancies between the current and previous studies may have to do with our reduced sample size and different diagnostic equipment but also with patient characteristics. However, it is noteworthy that most of the SCI patients in our sample had few or no anatomical risk factors, since they were young and thin (none of them were obese, based on their BMI, and only two had overweight). This suggests that the increased occurrence of SDB in SCI patients is associated with the injury and highlights the need for formal sleep studies to diagnose and treat sleep disorders in these patients.

The analysis of audio and SpO_2_ signals showed that SCI patients had more apneas, hypopneas, and desaturations than age-matched control subjects (Table 3). First, the automatic detection of SEv from audio signals allowed us to calculate the AHI, which was found to be significantly higher in the SCI group, indicating their increased risk of SDB. Then, the classification of apneas and hypopneas permitted the investigation of which kind of events were more common in each patient and to show that SCI patients had an increased number of both apneas and hypopneas (Figure 3b). Respiratory dysfunction and impaired respiratory muscle strength are factors that may explain the increased occurrence of both apneas and hypopneas in SCI patients [16].

It is noteworthy that, although the AHI, AI, HI, ODI, CT90, and CT94 were significantly higher in the SCI than in the control group, there were no differences in awake SpO_2_ or in median SpO_2_ (Table 3). Therefore, SCI patients had more desaturations than control subjects and reached lower minimum SpO_2_ values, but this did not induce significant changes in median SpO_2_ levels during sleep or in awake oxygen saturation. If confirmed in a larger study, the lack of significant differences in these parameters could suggest a different oxygenation pattern in SCI patients than controls, with a reduced effect of desaturations in the median SpO_2_ levels. On the other hand, although CT90 was significantly higher in SCI patients than controls, CT90 values of most SCI patients were relatively low considering their elevated ODI and AHI (Table 2). For instance, a study on nocturnal oximetry in SCI individuals, defining the hypoxia threshold when >10% of the time overnight was spent with SpO_2_ < 90% (i.e., CT90 > 10%), reported that 3 of the 10 patients (30%) met the criteria [47]. In our sample, only 4 of the 19 SCI patients (21%) had CT90 > 10%, which is not much higher than in control subjects (3 out of 19: 16%), despite the much higher number of apneas and hypopneas in SCI patients.

In addition to evaluating SDB according to the AHI and oxygen saturation parameters, we analyzed the spectral content of audio signals to identify nasal and oral breathing [32] throughout the night and calculated the percentage of time that each patient was breathing through the mouth. We found that the amount of oral breathing was remarkably higher in SCI individuals when compared to the control sample (Table 3, Figure 3c). To our knowledge, this is one of the first works investigating breathing route during sleep in SCI patients. Breathing route influences upper airway collapsibility since oral breathing dries the airways and increases their resistance [48,49]. This explains why excessive mouth breathing has been related to an increased risk and severity of obstructive sleep apnea in able-bodied subjects [50,51]. Moreover, monitoring oral breathing may have other purposes, as it is also indicative of nasal congestion, allergies, and other medical conditions [52]. Patients with tetraplegia often report nasal congestion, and nasal resistance was found to be elevated in these patients, potentially contributing to the high prevalence of SDB in this population [53]. This might be a reason for the high amount of oral breathing found in the SCI participants in our study. Therefore, percentage of oral breathing may serve as a surrogate measure of nasal congestion, and it can be easily monitored in a non-invasive way using the built-in microphones of smartphones. This information could support personalized therapeutic interventions to improve respiratory function and sleep quality in SCI individuals.

We investigated the correlations of the outcome variables with age, BMI, and the injury characteristics. Both age and BMI were strongly correlated with AHI, ODI, and other SDB measures, indicating that, in our sample, older patients and patients with higher BMI had more apneas, hypopneas, and desaturations (Figure 5a,b). Significant correlations were also found between age and CT94 and CT90, indicating lower SpO_2_ levels in older patients. Of note is the fact that, in our sample, all patients under 35 years of age had AHI < 15, i.e., mild SDB (the lowest severity). A similar finding was obtained by Stockhammer et al., who observed that all patients below 40 years old (11 out of 50) had AHI < 10 [14]. Age and BMI are both known risk factors for SDB in able-bodied subjects, but their relationship with SDB in SCI patients is not so clear [9]. While some studies in SCI have noted the correlation between SDB and increased age [14,54,55] and BMI [14,27,47], others failed to find any association [11,56], possibly because of the overrepresentation of young and non-obese patients in studies of SCI [9,14]. We could not investigate the relationship between SDB and sex, because our sample included only three women with SCI (16%). It is difficult to recruit female SCI patients for research studies, since the incidence of SCI is higher in males (male:female ratios range from 1.1:1 to 7:1 in developed countries [57]).

Regarding injury characteristics, AHI was not found to be correlated with injury level, completeness (AIS), or time post-injury. The lack of correlation between AHI and injury level may be because the five patients with thoracic injuries—two of whom had the highest BMI—had high AHI (three moderate SDB and two severe SDB). For this reason, when considering only the subset of patients with cervical injuries, the AHI significantly correlated with injury level, suggesting that patients with higher injuries suffered more severe SDB (Figure 5c). In addition, the minimum SpO_2_ was correlated with injury level in the entire sample, showing that patients with higher injuries reached lower SpO_2_ levels due to the desaturations (Figure 5d). On the other hand, we could not properly study the correlation with AIS level since most of the youngest patients had complete injuries and, thus, age was acting as a confounding factor. There are contrasting results in the literature regarding the relationship between injury characteristics and SDB [9]. Some previous studies associate higher lesions in tetraplegic patients with SDB [25,29,47], but in most cases no correlation was found with the injury level [11,12,13,46,55]. Similar contradictory findings have been found for the level of completeness: while some studies found a higher SDB occurrence in patients with complete [29] or incomplete injuries [25], most of them did not find relationships between SDB and the level of completeness [14,27,46]. As in our case, most of these studies are limited by the low number of patients (especially paraplegic patients), which could explain the discrepancies and low predictive power. In our study, we are aware that the sample was too small and heterogeneous to extract meaningful conclusions from the correlation analysis. However, this was not a population study but rather a proof-of-concept of the feasibility of smartphones for monitoring SDB in SCI individuals. The recruitment of patients with SCI for sleep studies remains a challenge due to the fact of their special health needs and the low incidence of SCI compared to other health conditions, which explains why most scientific publications related to this disorder involve small sample sizes [58].

Triaxial accelerometer data were also acquired by the smartphone during the overnight recordings, which allowed us to monitor sleep position with angular resolution and to investigate the association between sleep position and the occurrence of apnea/hypopnea events using the algorithms previously developed by our group [34]. While classic techniques for sleep position monitoring, including PSG, classify sleep position into only four different categories (i.e., supine, right, left, and prone), our system permits calculating the sleep angle, thus increasing the resolution to provide a more accurate detection of sleep position. In this study, for the first time, we monitored the sleep angle throughout the night in SCI individuals, obtaining representations such as those in Figure 4a–d. As expected because of their motor disability, SCI patients slept predominantly in supine-like positions and moved less than control subjects. In most tetraplegic patients, only a few position changes occurred during the night, when their position in bed was changed by medical staff to prevent pressure sores.

In the able-bodied population, supine position is known to be a risk factor for SDB, since it may promote the occurrence of apneic events, a phenomenon that is known as positional sleep apnea. In SCI individuals, very few works have documented sleep position and investigated its association with SDB. McEvoy et al. conducted a sleep study in 40 tetraplegic patients, treating posture as a dichotomous variable (supine posture vs. other postures) and reported that AHI was higher in patients who slept supine compared with those in other postures [12]. Here, we investigated the relationship between sleep position and the occurrence of apnea/hypopnea events in SCI patients, obtaining for each participant the distribution of sleep position and the distribution of events along each specific sleep angle (Figure 4c–f). With these representations, it was possible to identify if there was a range of sleep angles where the occurrence of events was much higher than the percentage of time spent at that position. Therefore, we could distinguish between patients with positional sleep apnea (those with an increased frequency of events in supine positions, Figure 4c–e) and patients with non-positional behavior (Figure 4d–f). While most of the control subjects with AHI > 5 (9 of 14, 64%) had positional sleep apnea, a clear positional behavior was only observed in 8 of the 19 SCI patients (42%). However, due to the reduced number of sleep position changes in these patients because of their paralysis, it was difficult to assess the association between sleep angle and the occurrence of events. Future studies with larger sample sizes would be required to further investigate the impact of supine position on the risk of SDB in SCI individuals. Nevertheless, thanks to this kind of analysis, we can obtain personalized information for each patient and identify which patients suffer from positional sleep apnea. Since most SCI patients require assistance for changing posture during the night, this information can be valuable for clinicians to know which range of sleep angles to avoid in order to reduce sleep-related problems. Furthermore, in able-bodied snorers, nasal resistance is higher in the supine position compared with upright [59], so avoiding supine position could help to deal with the excessive mouth breathing of SCI patients.

As indicated by the results of this paper and a large amount of previous literature, SDB is a serious secondary problem in individuals with SCI, which can cause difficulty in the recovery of these patients, contribute to poor health outcomes, and compromise quality of life. Yet, access to diagnosis and management of sleep disorders remains limited for SCI patients [15], given their health issues but also the limitations in the current diagnostic system. PSG, the gold-standard technique, is costly and complex, and most sleep laboratories are not prepared to address the needs of paraplegic and tetraplegic patients. In this paper, we presented a simple unobtrusive tool for sleep apnea monitoring using the sensors of a smartphone. This portable system can easily be implemented in clinical settings, either in hospital or at home, to facilitate the assessment of SDB in SCI patients.

This work is a proof-of-concept demonstrating the feasibility of a new smartphone-based technology to record three signals of high clinical value (audio, SpO_2_, and accelerometry) and provide information for overnight sleep monitoring in SCI patients. In our study, the smartphone system, as a simple non-invasive tool, facilitated the sleep recordings in paraplegic and tetraplegic patients. No problems were encountered during the overnight recordings, and none of the patients reported any side effect or discomfort. Yet, we must remark that as the proposed device is still under research and is not a standard clinical procedure such as conventional PSG, the results regarding SDB incidence in SCI patients must be considered cautiously. However, all the algorithms presented here for sleep position monitoring and for apnea/hypopnea detection and classification have been validated in previous works, demonstrating a good agreement with either PSG or a commercially available type III portable device for home sleep apnea diagnosis [32,34]. Specifically, the AHI estimation from the audio signals recorded with the smartphone showed a 99% concordance with the reference device, with an accuracy for apnea/hypopnea classification of 82% [32], while sleep position monitoring demonstrated a 96% agreement with video-validated position from PSG [34]. Nonetheless, it is worth mentioning some limitations of this study and possible future extensions. First, despite the already high SDB severity found in SCI patients, the AHI might be slightly underestimated for several reasons: (1) hypopneas with snoring could be missed since our algorithm was specifically designed to detect silence events [32], and (2) as the apnea/hypopnea detection is guided by SpO_2_ (i.e., only regions preceding desaturations are analyzed), we could miss apneas not followed by desaturations or hypopneas associated with arousals. To address these issues, in future work we could combine audio information with other channels from the smartphone or external sensors but also implement machine learning and deep learning approaches and compare them with the proposed rule-based algorithms. On the other hand, future extensions could include increasing the sample size to better assess the effects of the injury characteristics (injury level, completeness, and time post-injury) and other factors (including medication use or rehabilitation treatments) into SDB severity in SCI patients. Longitudinal studies could also be useful to investigate AHI variability between different nights, follow up the patient’s condition, and even assess the effects of rehabilitation. These studies would be extremely costly with current procedures. However, the proposed smartphone system is a simple non-invasive tool which would reduce the costs and complexity of medical sleep monitoring. Therefore, it could facilitate access to sleep studies for SCI patients to improve the detection and management of SDB in these patients with subsequent benefits for their overall health.

## 5. Patents

The algorithms presented in this manuscript are under a process to recognize industrial property.

## Figures and Tables

**Table 1:** Clinical characteristics of the SCI individuals.

Patient ID	Gender	Age (Years)	BMI (kg/m^2^)	Injury Level	AIS	Months Post-Injury	Etiology
SCI 1	M	47	24.8	C4	A	6.6	Traumatic
SCI 2	F	27	20.8	C4	A	9.6	Traumatic
SCI 3	M	18	17.3	C4	A	11.9	Traumatic
SCI 4	M	47	22.0	C4	C	3.0	Traumatic
SCI 5	M	55	20.3	C4	C	5.7	Traumatic
SCI 6	M	45	20.7	C4	C	6.7	Traumatic
SCI 7	M	60	23.8	C4	D	2.8	Traumatic
SCI 8	M	76	22.2	C4	D	8.4	Traumatic
SCI 9	M	31	24.2	C5	A	7.7	Traumatic
SCI 10	M	19	23.2	C5	C	3.4	Non-traumatic
SCI 11	M	46	21.0	C6	A	5.0	Traumatic
SCI 12	M	67	23.5	C6	A	6.1	Traumatic
SCI 13	M	20	19.6	C6	B	6.2	Traumatic
SCI 14	M	38	20.6	C8	B	6.2	Traumatic
SCI 15	M	34	21.9	T7	B	1.7	Traumatic
SCI 16	F	55	28.8	T9	D	2.4	Non-traumatic
SCI 17	M	53	28.9	T10	B	3.7	Traumatic
SCI 18	M	43	22.2	T11	A	1.3	Traumatic
SCI 19	F	38	22.3	T11	D	4.1	Traumatic
Mean ± SD/ Total	16 M (84%) 3 F (16%)	43 ± 16	22.5 ± 2.8	14 cervical (C4–C8) 5 thoracic (T7–T11)	7 AIS A 4 AIS B 4 AIS C 4 AIS D	5.4 ± 2.8	Traumatic: 17 (89%) Non-traumatic: 2 (11%)

**Table 2:** Outcome measures for all SCI patients. Patients with mild sleep apnea (5 ≤ AHI < 15) are highlighted in yellow, patients with moderate sleep apnea (15 ≤ AHI < 30) in orange, and patients with severe sleep apnea (AHI ≥ 30) in red.

Patient ID	Awake SpO_2_	Median SpO_2_	Minimum SpO_2_	CT94 (%)	CT90 (%)	ODI (h^−1^)	AHI (h^−1^)	AI (h^−1^)	HI (h^−1^)	Time in Events (%)	Oral Breathing (%)
SCI 1	98	95	78	24.33	3.73	40.44	42.56	11.55	31.01	28.58	11.42
SCI 2	98	98	76	0.61	0.17	10.03	12.09	5.62	6.47	8.65	67.91
SCI 3	98	95	78	23.30	3.18	14.82	13.85	9.84	4.01	6.76	31.03
SCI 4	95	95	54	32.15	15.21	59.59	60.05	30.40	29.64	31.24	46.57
SCI 5	95	94	73	44.11	5.55	17.66	15.59	3.29	12.30	7.21	12.11
SCI 6	99	92	74	70.38	24.41	20.83	24.84	8.93	15.91	19.68	8.81
SCI 7	95	95	83	26.73	1.98	43.00	45.85	21.32	24.54	37.29	22.73
SCI 8	92	90	81	98.41	39.04	25.38	29.43	9.29	20.14	19.91	61.64
SCI 9	98	96	85	7.05	0.06	14.06	14.54	3.60	10.93	8.10	44.77
SCI 10	96	93	61	69.88	0.82	14.00	8.50	1.87	6.62	3.46	63.72
SCI 11	98	95	81	4.95	0.45	11.49	11.10	9.64	1.45	5.20	52.63
SCI 12	98	94	74	44.35	9.87	30.74	35.19	12.20	22.99	24.58	55.87
SCI 13	99	95	90	13.13	0.00	9.02	8.77	1.98	6.79	6.37	36.29
SCI 14	97	95	88	6.62	3.23	7.59	9.06	4.53	4.53	7.21	11.24
SCI 15	94	95	88	7.20	0.15	16.09	16.33	3.87	12.46	9.20	34.79
SCI 16	94	90	80	89.95	37.78	26.60	30.42	10.56	19.85	21.57	63.42
SCI 17	96	95	80	22.68	1.11	39.98	42.17	26.10	16.07	28.64	43.35
SCI 18	99	95	89	14.75	0.05	25.01	24.89	15.13	9.76	20.32	30.75
SCI 19	95	94	79	44.91	1.10	22.44	28.60	15.08	13.51	19.48	12.96

**Table 3:** Group means, standard deviations, and ranges for each variable for the SCI and control groups.

Group	Statistic	Awake SpO_2_	Median SpO_2_	Minimum SpO_2_	CT94 (%)	CT90 (%)	ODI (h^−1^)	AHI (h^−1^)	AI (h^−1^)	HI (h^−1^)	Time in Events (%)	Oral Breathing (%)
SCI	Mean ± SD	97 ± 2	94 ± 2	79 ± 9	34 ± 29	8 ± 12	24 ± 14	25 ± 15	11 ± 8	14 ± 9	16 ± 10	37 ± 20
	Range	92–99	90–98	54–90	0.6–98	0–39	8–60	8–60	2–30	1–31	3.5–37	9–68
Control	Mean ± SD	96 ± 2	94 ± 2	86 ± 8	25 ± 36	4 ± 11	9 ± 7	9 ± 7	5 ± 3	5 ± 5	5 ± 4	11 ± 16
	Range	93–99	90–98	60–95	0–95	0–39	0.7–27	0.3–24	0.3–9	0–19	0.1–13	1.3–66
*p*-Value		0.26	0.77	0.003	0.04	0.003	<0.001	<0.001	0.006	<0.001	<0.001	<0.001

## Data Availability

The data presented in this study are available on request from the corresponding authors.

## Abbreviations

AASM	American Academy of Sleep Medicine
AI	Apnea Index
AIS	American Spinal Injury Association Impairment Scale
AHI	Apnea–Hypopnea Index
BMI	Body Mass Index
CBCT	Cone-Beam Computed Tomography
CT90	Cumulative Time with SpO_2_ below 90%
CT94	Cumulative Time with SpO_2_ below 94%
FFT	Fast Fourier Transform
fSampEn	Fixed Sample Entropy
HI	Hypopnea Index
mHealth	Mobile Health
MMP	Modified Mallampati
ODI	Oxygen Desaturation Index
PSG	Polysomnography
RMS	Root Mean Square
SCI	Spinal Cord Injury
SD	Standard Deviation
SDB	Sleep-Disordered Breathing
SEv	Silence Event
SNR	Signal-to-Noise Ratio
SpO_2_	Oxygen Saturation
